# Economic Evaluation of Ticagrelor Plus Aspirin Versus Aspirin Alone for Acute Ischemic Stroke and Transient Ischemic Attack

**DOI:** 10.3389/fphar.2022.790048

**Published:** 2022-03-18

**Authors:** Jigang Chen, Linjin Ji, Xin Tong, Mingyang Han, Songfeng Zhao, Yongkai Qin, Zilong He, Zhiqun Jiang, Aihua Liu

**Affiliations:** ^1^ Department of Interventional Neuroradiology, Beijing Tiantan Hospital, Capital Medical University, Beijing, China; ^2^ Beijing Neurosurgical Institute, Capital Medical University, Beijing, China; ^3^ Department of Neurosurgery, The First Affiliated Hospital of Nanchang University, Nanchang, China; ^4^ Department of Neurosurgery, The Third Xiangya Hospital, Central South University, Changsha, China; ^5^ China National Clinical Research Centre for Neurological Diseases, Beijing, China

**Keywords:** ticagrelor, aspirin, stroke, transient ischemic attack, cost-effectiveness analysis

## Abstract

**Background:** Although ticagrelor plus aspirin is more effective than aspirin alone in preventing the 30-day risk of a composite of stroke or death in patients with an acute mild-to-moderate ischemic stroke (IS) or transient ischemic attack (TIA), the cost-effectiveness of this combination therapy remains unknown. This study aims to determine the cost-effectiveness of ticagrelor plus aspirin compared with aspirin alone.

**Methods:** A combination of decision tree and Markov model was built to estimate the expected costs and quality-adjusted life-years (QALYs) associated with ticagrelor plus aspirin and aspirin alone in the treatment of patients with an acute mild-to-moderate IS or TIA. Model inputs were extracted from published sources. One-way sensitivity, probabilistic sensitivity, and subgroup analyses were performed to test the robustness of the findings.

**Results:** Compared with aspirin alone, ticagrelor plus aspirin gained an additional lifetime QALY of 0.018 at an additional cost of the Chinese Yuan Renminbi (¥) of 269, yielding an incremental cost-effectiveness ratio of ¥15,006 (US$2,207)/QALY. Probabilistic sensitivity analysis showed that ticagrelor plus aspirin had a probability of 99.99% being highly cost-effective versus aspirin alone at the current willingness-to-pay threshold of ¥72,447 (US$10,500)/QALY in China. These findings remain robust under one-way sensitivity and subgroup analyses.

**Conclusions:** The results indicated that early treatment with a 30-days ticagrelor plus aspirin for an acute mild-to-moderate IS or TIA is highly cost-effective in a Chinese setting.

## Introduction

The risk of another ischemic stroke (IS) occurring after an acute mild-to-moderate IS or transient ischemic attack (TIA) is very high, and nearly 5%–10% of patients would have a stroke in the first few months (Johnston et al., 2000; Giles and Rothwell, 2007). Aspirin has been used for secondary stroke prevention among these patients with only modest benefits (Chen, 1997; International Stroke Trial Collaborative Group, 1997). Two trials show that adding clopidogrel to aspirin is superior to aspirin alone in reducing the risk of stroke and other major ischemic events (Wang et al., 2013; Johnston et al., 2018). Yet, the efficacy of clopidogrel varies among individuals with different metabolic activations due to the reduced function of cytochrome *CYP2C19*, and a considerable number of strokes still occur even with clopidogrel or dual antiplatelet therapy (Pan et al., 2017).

Ticagrelor is a platelet aggregation inhibitor that reversibly binds and inhibits the P2Y_12_ receptor. It has the advantage of being unaffected by *CYP2C19* polymorphisms. The Acute Stroke or Transient Ischemic Attack Treated with Ticagrelor and ASA (acetylsalicylic acid) for Prevention of Stroke and Death (THALES) trial showed that early treatment with ticagrelor plus aspirin for the first 30 days was superior to aspirin alone in reducing the 30-day risk of a composite of stroke or death [5.5% vs. 6.5%; hazard ratio (HR), 0.83; 95% confidence interval (CI), 0.71–0.96] (Johnston et al., 2020). Adding ticagrelor to aspirin also reduced the total burden of disability (odds ratio, 0.77; 95% CI, 0.65–0.91) owing to IS recurrence (Amarenco et al., 2021). However, the incidence of severe bleeding was significantly higher in the ticagrelor–aspirin group (0.5% vs. 0.1%; HR, 3.99; 95% CI, 1.74–9.14) (Johnston et al., 2020).

Although the combination of ticagrelor and aspirin could reduce the risk of a composite of stroke or death, it is associated with higher adverse events and higher costs when compared with aspirin alone. Therefore, medical decision analysis is needed to evaluate the advantage or disadvantage of ticagrelor plus aspirin over aspirin alone. Currently, the best method of doing this is cost-effectiveness analysis, which aims to assess the overall costs of different drugs and treatment procedures as well as the overall effectiveness related to different outcomes. In this study, we aim to determine the cost-effectiveness of adding ticagrelor to aspirin in patients with an acute mild-to-moderate IS or TIA.

## Methods

### Model Overview

**FIGURE 1:**
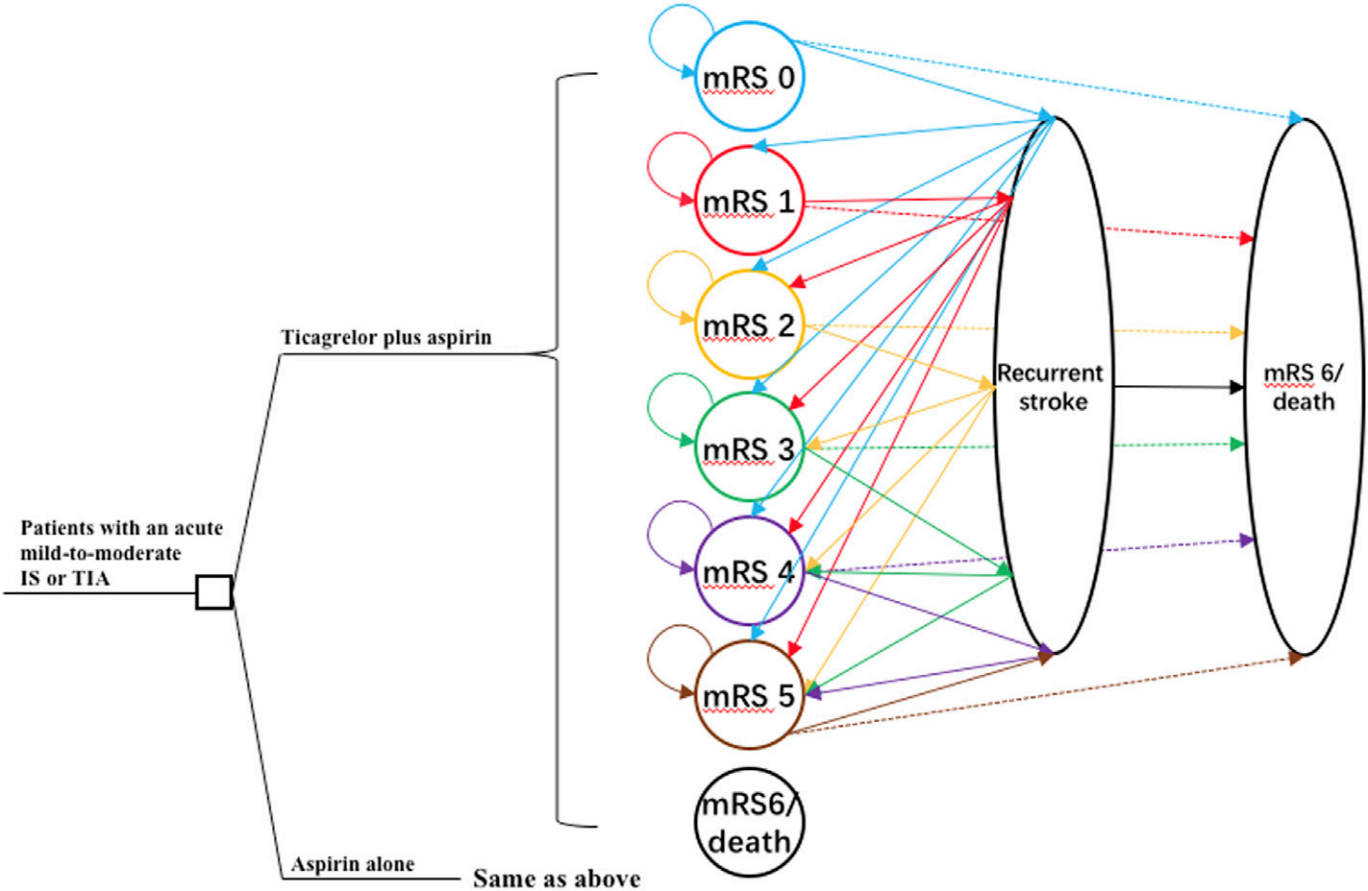
The schematic structure of the model. A patient with an acute mild-to-moderate IS or TIA entered the model at 65 years old receiving either ticagrelor plus aspirin or aspirin alone for the first 30 days. Patients would distribute among different health statuses determined by mRS scores at 30 days and transit to a state of equal or greater disability after recurrent stroke or die after 30 days. IS, ischemic stroke; mRS, modified Rankin Scale; TIA, transient ischemic attack.

This study was conducted according to the Consolidated Health Economic Evaluation Reporting Standards (CHEERS) reporting guidelines (Husereau et al., 2013). A combination of decision tree and Markov model was developed using TreeAge Pro 2020 software (Tree Age Software, Inc., One Bank Street, Williamstown, MA, United States of America) to estimate the long-term costs and outcomes of two antiplatelet therapies: 1) ticagrelor plus aspirin therapy: a loading dose of 180 mg ticagrelor (given as two 90-mg tablets) followed by a maintenance dose of 90 mg ticagrelor twice daily on day 2 to day 30 plus a loading dose of 300 mg aspirin on day 1 followed by a maintenance dose of 75–100 mg aspirin daily on day 2 to day 30; 2) aspirin-alone therapy: a loading dose of 300 mg aspirin on day 1 followed by a maintenance dose of 75–100 mg aspirin daily on day 2 to day 30. The target population was analogous to that of the THALES trial (Johnston et al., 2020). Patients were 65 years old on average. They had either an acute mild-to-moderate IS or TIA and were not undergoing intravenous or endovascular thrombolysis. Patients in the two treatment arms entered the Markov model at the health state of modified Rankin scale (mRS) score of 0 and transited to other health states including mRS 1, 2, 3, 4, 5, and 6 (death) in the next cycle. The occurrence of adverse events such as IS, intracranial hemorrhage (ICH), and major extracranial hemorrhage (ECH), as defined according to the Global Utilization of Streptokinase and Tissue Plasminogen Activator for Occluded Coronary Arteries trial (Gusto Investigators, 1993), was incorporated into the model with additional costs and disutility. The cycle length was 1 month, and the time horizon was 30 years. The schematic structure of the model is provided in Figure 1.

### Input Parameters

Input parameters of this model were obtained from the THALES trial (Johnston et al., 2020; Amarenco et al., 2021) and the most recently published literature if possible (Table 1). In the aspirin-alone group, the probability of a primary outcome (the composite of stroke or death) in the first 30 days was 0.066. In the ticagrelor-plus-aspirin group, the probability of primary outcome was estimated based on the HR (0.83) between these two groups. The proportion of death, IS, and ICH among patients with primary outcome and the probability of major ECH were extracted according to their respective event data reported by the THALES trial (Johnston et al., 2020).

**TABLE 1:** List of input parameters.

Parameters	Base case value	Range	Distribution	Source
30-day outcome of aspirin alone				
Probability of primary outcome	0.066	0.060–0.073	Beta, SD: 0.003	Johnston et al. (2020)
Proportion of death	0.075	0.052–0.106	Beta, SD: 0.014	Johnston et al. (2020)
Proportion of IS	0.953	0.926–0.970	Beta, SD: 0.011	Johnston et al. (2020)
Proportion of ICH	0.017	0.008–0.036	Beta, SD: 0.007	Johnston et al. (2020)
Probability of major ECH	0.001	0.000–0.003	Beta, SD: 0.001	Johnston et al. (2020)
Proportion of mRS 0	0.365	—	—	Amarenco et al. (2021)
Proportion of mRS 1	0.395	—	—	Amarenco et al. (2021)
Proportion of mRS 2	0.140	—	—	Amarenco et al. (2021)
Proportion of mRS 3	0.056	—	—	Amarenco et al. (2021)
Proportion of mRS 4	0.034	—	—	Amarenco et al. (2021)
Proportion of mRS 5	0.004	—	—	Amarenco et al. (2021)
30-day outcome of ticagrelor added to aspirin				
HR of primary outcome	0.830	0.710–0.960	Beta: SD: 0.060	Johnston et al. (2020)
Proportion of death	0.119	0.087–0.160	Beta, SD: 0.018	Johnston et al. (2020)
Proportion of IS	0.911	0.874–0.938	Beta, SD: 0.016	Johnston et al. (2020)
Proportion of ICH	0.066	0.043–0.100	Beta, SD: 0.014	Johnston et al. (2020)
Probability of major ECH	0.005	0.004–0.007	Beta, SD: 0.001	Johnston et al. (2020)
Proportion of mRS 0	0.372	—	—	Amarenco et al. (2021)
Proportion of mRS 1	0.390	—	—	Amarenco et al. (2021)
Proportion of mRS 2	0.139	—	—	Amarenco et al. (2021)
Proportion of mRS 3	0.057	—	—	Amarenco et al. (2021)
Proportion of mRS 4	0.031	—	—	Amarenco et al. (2021)
Proportion of mRS 5	0.004	—	—	Amarenco et al. (2021)
Probabilities				
Recurrent rate of stroke per life-year	0.122	0.116–0.128	Beta, SD: 0.003	Xu et al. (2007)
Proportion of ICH	0.075	0.075–0.146	Beta, SD: 0.018	Johnston et al. (2020)
RR of stroke recurrence per life-year	1.030	1.020–1.040	Lognormal, SD: 0.005	Pennlert et al. (2014)
Death after recurrent stroke	0.193	0.174–0.213	Beta, SD: 0.010	Xu et al. (2007)
Mortality hazard ratios				
mRS 0	1.000	—	Lognormal, SD: 0.050	Samsa et al. (1999)
mRS 1	1.000	—	Lognormal, SD: 0.050	Samsa et al. (1999)
mRS 2	1.110	—	Lognormal, SD: 0.083	Samsa et al. (1999)
mRS 3	1.270	—	Lognormal, SD: 0.127	Samsa et al. (1999)
mRS 4	1.710	—	Lognormal, SD: 0.171	Samsa et al. (1999)
mRS 5	2.370	—	Lognormal, SD: 0.237	Samsa et al. (1999)
Cost (2020 Chinese Yuan Renminbi, ¥)				
Additional cost of ticagrelor	394	174–593	Gamma, SD: 105	Tuling
Hospitalization cost for IS, independent	10,958	13,698–8,219	Gamma, SD: 1370	Wang et al. (2018), Pan et al. (2020)
Hospitalization cost for IS, dependent	13,605	10,204–17,006	Gamma, SD: 1701	Wang et al. (2018), Pan et al. (2020)
Hospitalization cost for IS, death	11,970	8,978–14,963	Gamma, SD: 1496	Wang et al. (2018), Pan et al. (2020)
Hospitalization cost for ICH, independent	13,174	9,881–16,468	Gamma, SD: 1647	Pan et al. (2014)
Hospitalization cost for ICH, dependent or death	17,490	13,118–21,863	Gamma, SD: 2186	Pan et al. (2014)
Hospitalization cost for major ECH	8,535	6,401–10,669	Gamma, SD: 1067	Pan et al. (2014)
Annual posthospitalization cost for independent	8,310	6,233–10,388	Gamma, SD: 1039	Pan et al. (2018)
Annual posthospitalization cost for dependent	12,771	9,578–15,964	Gamma, SD: 1596	Pan et al. (2018)
Utility				
mRS 0	0.850	0.800–1.000	Beta, SD: 0.050	Gage et al. (1998), Earnshaw et al. (2009), Nelson et al. (2016), Peultier et al. (2020)
mRS 1	0.800	0.800–0.950	Beta, SD: 0.038	Gage et al. (1998), Earnshaw et al. (2009), Nelson et al. (2016), Peultier et al. (2020)
mRS 2	0.700	0.680–0.900	Beta, SD: 0.055	Gage et al. (1998), Earnshaw et al. (2009), Nelson et al. (2016), Peultier et al. (2020)
mRS 3	0.510	0.450–0.650	Beta, SD: 0.050	Gage et al. (1998), Earnshaw et al. (2009), Nelson et al. (2016), Peultier et al. (2020)
mRS 4	0.300	0.100–0.400	Beta, SD: 0.075	Gage et al. (1998), Earnshaw et al. (2009), Nelson et al. (2016), Peultier et al. (2020)
mRS 5	0.150	0.000–0.320	Beta, SD: 0.080	Gage et al. (1998), Earnshaw et al. (2009), Nelson et al. (2016), Peultier et al. (2020)
mRS 6 or death	0.000			
Disutility of recurrent stroke	0.660	0.640–0.680	Beta, SD: 0.010	Ganesalingam et al. (2015)
Disutility of major ECH	0.200	0.160–0.230	Beta, SD: 0.018	Pan et al. (2014)

ECH, extracranial hemorrhage; HR, hazard ratio; ICH, intracranial hemorrhage; IS, ischemic stroke; mRS, modified Rankin scale; RR, relative risk; SD, standard deviation

All the patients were assumed to enter the model in the state of mRS 0, and they would be distributed to different states from mRS 0 to mRS 6 at the end of the first month after receiving ticagrelor plus aspirin or aspirin alone. The proportion of patients in different health states at the end of the first month was obtained from the THALES trial and has been provided in Table 1 (Amarenco et al., 2021).

After the initial month, the proportion of patients distributed to different health states was decided by the recurrent rate of stroke and the age-specific non-stroke death rates. The recurrent rate of stroke after the first 30 days was estimated from the China National Stroke Registry (CNSR) (Xu et al., 2007), and we assumed that the risk of stroke recurrence would increase by 1.03-fold per life-year (Pennlert et al., 2014). The death rate after recurrent stroke was reported to be 0.1933 (Xu et al., 2007), and patients who remained alive were assumed to be reallocated equally among health states of equal and greater disability (Pan et al., 2014; Peultier et al., 2020).

We obtained the age-specific non-stroke death rates from the most recent published census of China and adjusted the rates according to the causes of death in 2018 reported in the China Health Statistics Yearbook 2019 (National Bureau of Statistics of China, 2021a; National Health Commission of the People’s Republic of China, 2019). Dependent patients (mRS 3, 4, or 5) were reported to have increased mortality compared with independent patients (mRS 0, 1, or 2) (Slot et al., 2009), and we obtained mRS state-specific hazard ratios from previous reports (Samsa et al., 1999; Peultier et al., 2020). Patients who were alive and did not experience a recurrent stroke would remain in the same health state at the end of one cycle.

### Costs

This study was conducted from the perspective of Chinese healthcare payers, and only direct medical costs were included. The additional cost of ticagrelor was estimated according to the median retail price of ticagrelor from the widely used Chinese Drug Price database (Tuling, www.315jiage.cn). This database provides information about reference prices in different regions of China for the same drug. We validated the price of ticagrelor from this database with other famous Chinese online pharmacies as well as our institutional clinical database, and the prices were very close. One-time hospitalization costs for major events and posthospitalization costs were obtained from the most recent published studies conducted in China. To account for the uncertainty, a wide range of ±25% was used for all costs. Costs were converted to 2020 Chinese Yuan Renminbi (¥) according to the consumer price index (National Bureau of Statistics of China, 2020).

### Utility

Health-related quality of life value (utility scores) was assigned to all health states. Quality-adjusted life-years (QALYs) were calculated by multiplying the length period the patient spent in a particular state by the corresponding utility score. Utility scores for mRS 0, mRS 1, mRS 2, mRS 3, mRS 4, and mRS 5 were defined as 0.85 (0.8–1), 0.8 (0.8–0.95), 0.7 (0.68–0.9), 0.51 (0.45–0.65), 0.30 (0.1–0.4), and 0.15 (0–0.32), respectively (Gage et al., 1998; Earnshaw et al., 2009; Nelson et al., 2016; Peultier et al., 2020). Patients with recurrent stroke or major ECH were assumed to have a disutility of 0.66 and 0.2, respectively (Pan et al., 2014). All costs and utilities were discounted by 3% per year (Weinstein et al., 1996).

### Statistical Analysis

The primary measure in this study was the incremental cost-effectiveness ratio (ICER), which was defined as the incremental cost per additional QALY gained. One strategy was considered cost-effective when compared to another if the ICER was below the willingness-to-pay (WTP) threshold. As recommended by the World Health Organization, the WTP threshold was chosen as 1 × gross domestic product (GDP) per capita if one strategy was to be highly cost-effective. This WTP threshold corresponded to ¥72,447 (US dollars $10,500)/QALY in China in the year 2020 (National Bureau of Statistics of China, 2021b).

The base-case analysis was conducted using the mean value of all parameters. To identify key parameters related to the robustness of the results, one-way sensitivity analyses were performed by varying one parameter while keeping others fixed. To perform a probabilistic sensitivity analysis, all parameters were assigned with a distribution accordingly. These parameters varied simultaneously in the probabilistic sensitivity analysis with Monte Carlo simulation (10,000 iterations) to evaluate the impact of uncertainty. Moreover, subgroup analyses were performed in the prespecified subgroups as defined in the THALES trial by varying the HRs of primary outcomes between two antiplatelet therapy groups. The mean and range for these HRs were obtained from the subgroups reported in this trial (Johnston et al., 2020).

## Results

### Base-Case Analysis

In the base-case scenario, patients treated with aspirin alone lived an average of 6.764 QALYs, incurring a cost of ¥147,122. Those treated with ticagrelor plus aspirin lived an average of 6.782 QALYs (that is, an additional 0.018 QALY), with a lifetime cost of ¥147,391 or an additional cost of ¥269. The ICER for ticagrelor-plus-aspirin therapy relative to aspirin-alone therapy was ¥15,006 ($2,207)/QALY. Under the current threshold of ¥72,447/QALY, ticagrelor-plus-aspirin therapy was highly cost-effective in the base-case scenario.

### Sensitivity Analyses

**FIGURE 2:**
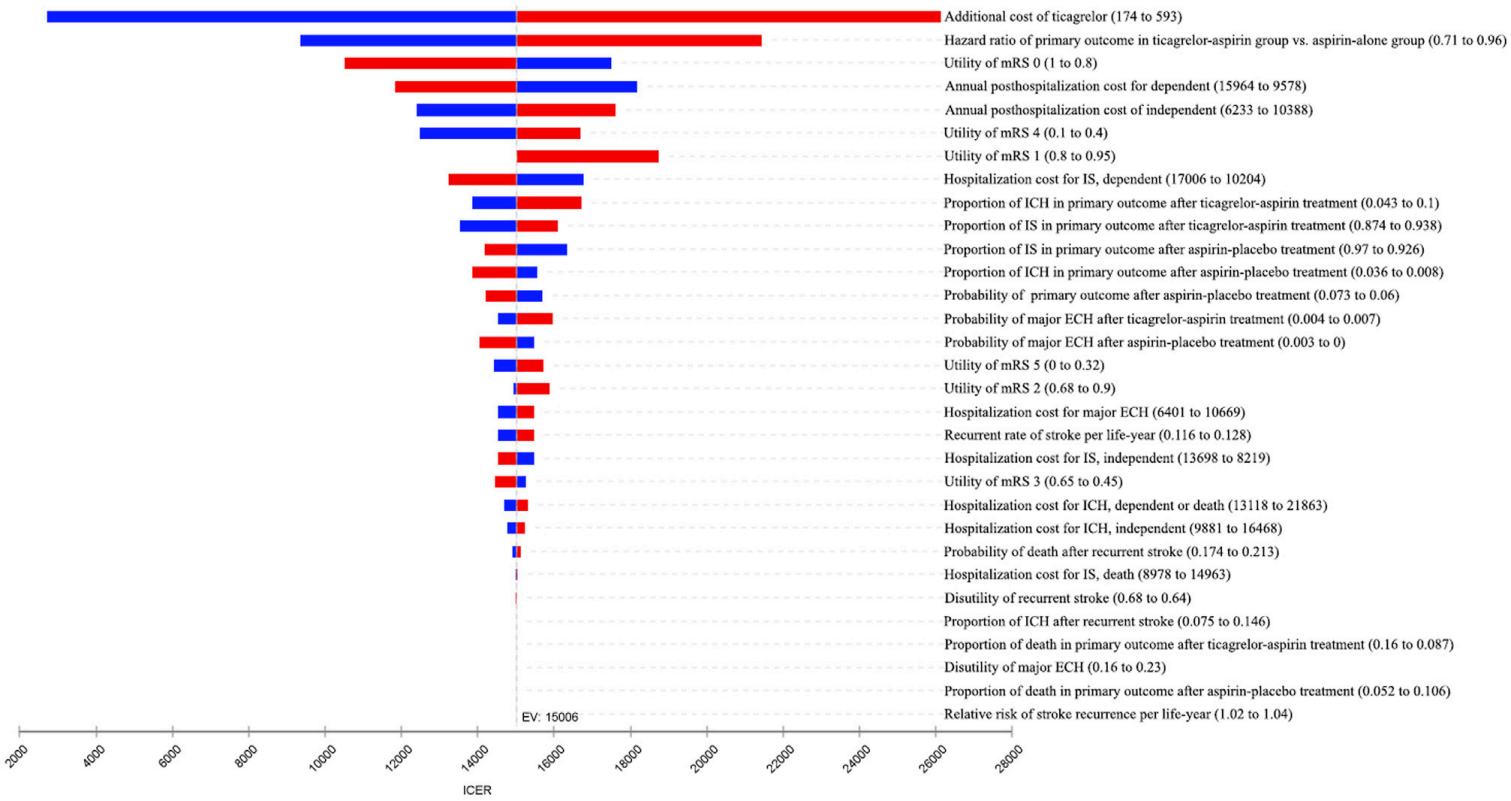
Tornado diagram of one-way sensitivity analyses. The plot shows how varying one input parameter to its limits at a time affects the incremental cost-effectiveness ratio (ICER). ECH, extracranial hemorrhage; EV, expected value; ICH, intracranial hemorrhage; IS, ischemic stroke; mRS, modified Rankin Scale.

One-way sensitivity analyses were conducted to account for the impact of the uncertainty of different parameters on the ICER, and the results were presented in the tornado diagram (Figure 2). Overall, the results were most sensitive to the additional cost of ticagrelor as well as HR of primary outcome between two antiplatelet therapy groups. When the additional cost of ticagrelor ranged between ¥174 and ¥593, the corresponding ICER was between ¥2,712/QALY and ¥26,127/QALY. When the HR ranged between 0.71 and 0.96, the corresponding ICER was between ¥9,354/QALY and ¥21,440/QALY. All the ICERs, including those obtained by other varying parameters, were below the WTP threshold, indicating that the study results were robust.

**FIGURE 3:**
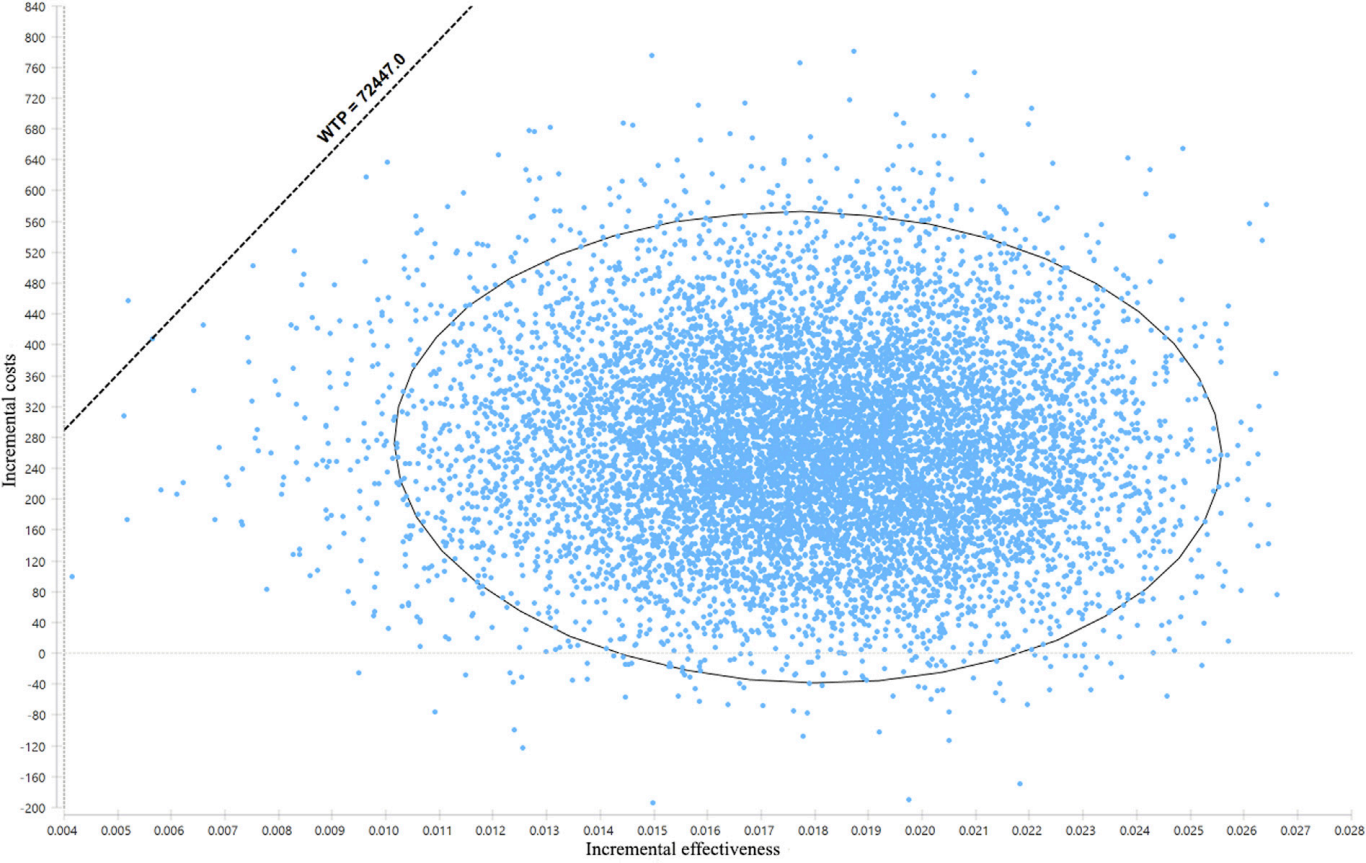
Results of the probabilistic sensitivity analysis. The dots that lie to the right of the willingness-to-pay (WTP) line mean the cases where ticagrelor plus aspirin is cost-effective when compared with aspirin alone.

The result of probabilistic sensitivity analysis is shown in Figure 3. Among the 10,000 simulation runs, ticagrelor-plus-aspirin therapy was superior in 99.99% of the simulations at a WTP threshold of ¥72,447/QALY.

### Subgroup Analyses

**FIGURE 4:**
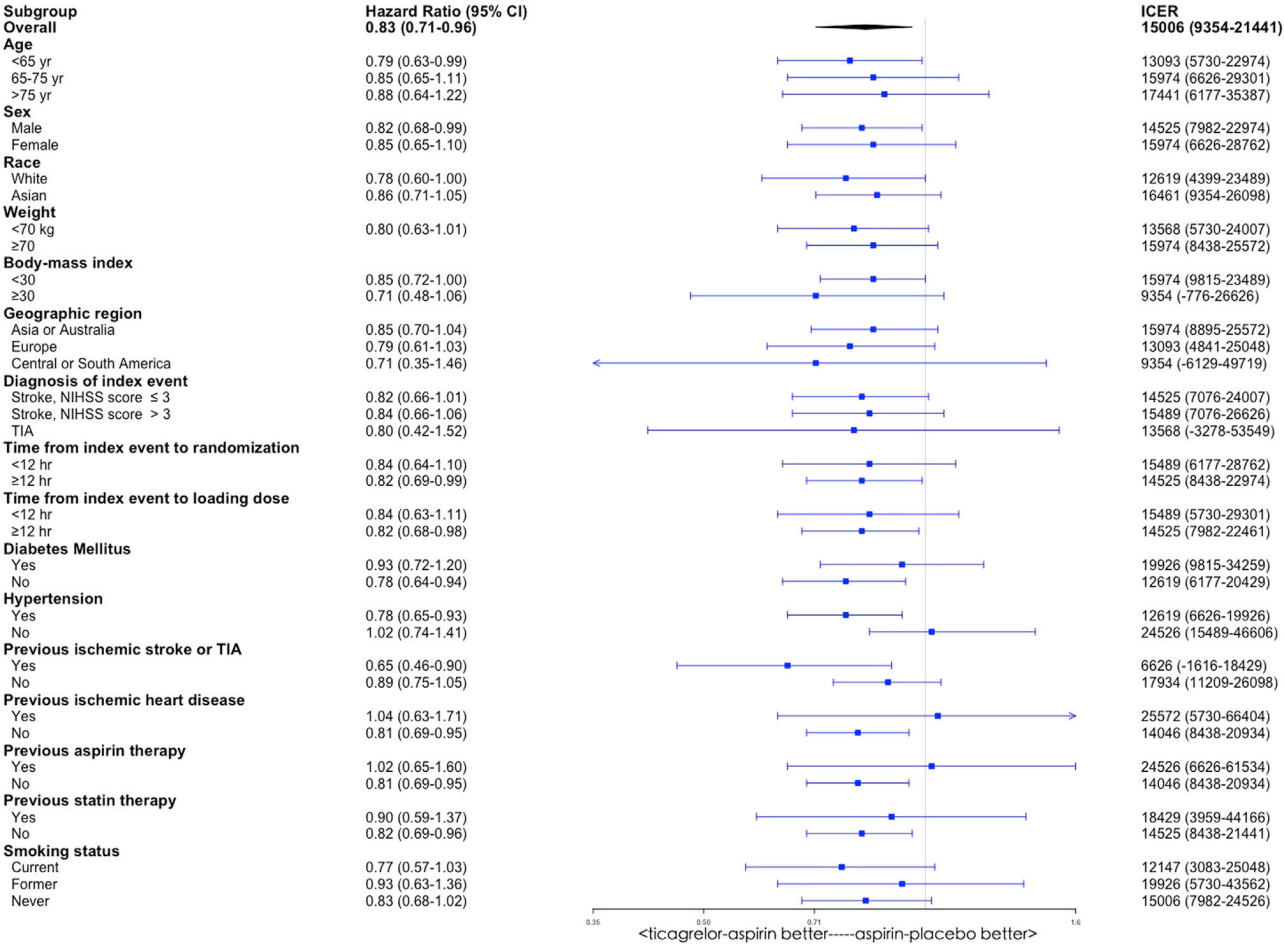
Subgroup analyses of incremental cost-effectiveness ratio (ICER) by varying the hazard ratio of primary outcome between the ticagrelor-plus-aspirin group and the aspirin-alone group. CI, confidence interval.

By varying the HRs for primary outcome between two antiplatelet therapy groups in the THALES trial subpopulations, subgroup analyses were conducted among the following subgroups including age, sex, race, weight, body mass index, geographic region, diagnosis of index event, time from index event to randomization, time from index event to loading dose, diabetes mellitus, hypertension, previous ischemic stroke or TIA, previous aspirin therapy, previous statin therapy, and smoking status. All the ICERs were below the WTP threshold, indicating that ticagrelor plus aspirin was cost-effective compared with aspirin alone in these subgroups (Figure 4).

## Discussion

For patients with an acute mild-to-moderate IS or TIA, adding ticagrelor to aspirin for 1 month increased life expectancy by 0.018 QALY over a lifetime, near 1 week of perfect health, at excellent value. This dual antiplatelet therapy gained an additional cost of ¥269, resulting in an ICER of ¥15,006/QALY. The robustness of our overall conclusion that ticagrelor-plus-aspirin therapy was cost-effective compared to aspirin-alone therapy was supported by the sensitivity and subgroup analyses. In the one-way sensitivity analyses, all the ICERs were below the WTP threshold when the input variables varied in their plausible ranges one by one. In the probabilistic sensitivity analysis, ticagrelor-plus-aspirin therapy was superior in 99.99% of simulations. Moreover, with the variations of HRs for the primary outcome, this dual antiplatelet therapy was favored in all the THALES trial subpopulations.

Our results were comparable to other similar studies. For example, the lifetime additional gain of QALY by ticagrelor-plus-aspirin therapy is 0.018 in the current study, while the lifetime QALY gain is 0.17 for clopidogrel when compared with aspirin for secondary prevention among stroke patients (Schleinitz et al., 2004) and 0.037 for clopidogrel plus aspirin when compared with aspirin alone (Pan et al., 2014). The gain of QALY associated with ticagrelor plus aspirin in our study is relatively smaller than other treatments. This is mainly because the 30-day incidence of disability did not differ significantly between the two groups in the THALES trial. Moreover, the incidence of adverse events such as major ECH was significantly higher when ticagrelor was added to aspirin, thus leading to a higher disutility of patients treated with this therapy. Notwithstanding, the comparable results between the current and other studies demonstrated the validity of our model.

To our knowledge, this study provides the first economic data on ticagrelor added to aspirin for the prevention of recurrent stroke. The advantage of this study is that we have utilized data from the THALES trial, which is a large-scale randomized trial that compares ticagrelor plus aspirin with aspirin alone directly. We conducted this study from the perspective of Chinese healthcare payers. There are over two million new cases of stroke in China every year, and it is related to the highest disability-adjusted life-years lost of any disease (Wu et al., 2019). Moreover, the stroke burden is expected to increase as a result of population aging and inadequate management. China has the largest population around the world, and there are fast-increasing demands for limited healthcare budgets. This drives policymakers to move towards a data-driven and evidence-supporting healthcare system with China’s national health strategy. Our cost-effectiveness study has the merits of providing an evidence-based reference regarding the secondary prevention practices for recurrent stroke.

A large body of studies has been published to assess the cost-effectiveness of acute stroke treatment and prevention in the last 2 decades. For acute ischemic stroke, intravenous alteplase is the recommended treatment, and investigators have evaluated its cost-effectiveness within different time windows after the stroke onset from the perspective of different countries including the United States, United Kingdom, China, and so on (Joo et al., 2017). These studies showed that intravenous alteplase was a dominant strategy compared with traditional treatment. Likewise, economic evaluation of mechanical thrombectomy, a recommended treatment for acute IS with a large vessel occlusion, has been increasingly conducted in recent years. According to a recent review, 25 studies from 12 different countries were published, and all these studies but one suggested that mechanical thrombectomy for stroke treatment was cost-effective (Waqas et al., 2021). The cost-effective evaluations regarding the long-term secondary prevention of IS with different drugs were published. These studies showed that clopidogrel, statin, warfarin, and dabigatran are regarded as the most cost-effective treatment for secondary stroke prevention. However, there is a lack of long-term outcome and resource use data, which adds great uncertainty to the cost-effectiveness (Pan et al., 2012).

Some limitations of our study should be noted. First, different treatment methods for IS were not incorporated into our model, while the mRS distribution of IS patients was significantly associated with treatment methods. However, we used the 30-day mRS scores as the post-treatment outcomes in our model as they were expected to be highly correlated with the post-treatment 90-day mRS scores (Rost et al., 2016). Moreover, the cost of IS treatment in the aspirin-alone group would be higher than the aspirin-plus-ticagrelor group, making aspirin alone less favorable. Second, our results were based on the efficacy findings of the THALES trial that was performed internationally, and the participants were mainly from Europe. It is unknown whether the combination therapy would show similar effects if the participants were restricted to Chinese patients. What is more, the utility scores were not Chinese population-specific. However, we have considered the difference in the sensitivity analyses, and the conclusion remains unchanged. Third, the one-time hospitalization costs and annual posthospitalization costs were different only between patients in independent and dependent status. Costs associated with different mRS scores were not obtained because no literature was available for these costs in China. Fourth, we assumed that patients who remained alive would be reallocated equally among health states of equal and greater disability. Dependent and independent patients were assumed to have the same probability of recurrent stroke. These assumptions might not reflect a real-world situation. However, they are not unprecedented in other cost-effectiveness studies (Pan et al., 2014; Peultier et al., 2020). Fifth, only direct costs were included in this analysis. If indirect and intangible costs such as loss of productivity were taken into consideration, it might produce a different result. Last, our model was built from the Chinese perspective and only reflected cost and event rates in China. Results might not accurately reflect the cost-effectiveness of ticagrelor added to aspirin in other countries.

## Conclusion

Early treatment with a 30-day ticagrelor plus aspirin for an acute mild-to-moderate IS or TIA is highly cost-effective in a Chinese setting. However, more studies are needed to evaluate the benefit and risks of this therapy.

## Data Availability

The original contributions presented in the study are included in the article/supplementary material; further inquiries can be directed to the corresponding authors.
